# Limits and Opportunities of SARS-CoV-2 Antigen Rapid Tests: An Experienced-Based Perspective

**DOI:** 10.3390/pathogens10010038

**Published:** 2021-01-05

**Authors:** Verena Schildgen, Sabrina Demuth, Jessica Lüsebrink, Oliver Schildgen

**Affiliations:** Institut für Pathologie, Kliniken der Stadt Köln, 51109 Cologne, Germany; demuths@kliniken-koeln.de (S.D.); luesebrinkj@kliniken-koeln.de (J.L.); schildgeno@kliniken-koeln.de (O.S.)

**Keywords:** rapid antigen tests, SARS-CoV-2, COVID-19

## Abstract

Background: Due to the steadily rising case numbers of SARS-CoV-2 infections worldwide, there is an increasing need for reliable rapid diagnostic devices in addition to existing gold standard PCR methods. Actually, public attention is focused on antigen assays including lateral flow tests (LFTs) as a diagnostic alternative. Therefore, different LFTs were analyzed regarding their performance in a clinical setting. Material and Methods: A pilot sample panel of 13 bronchoalveolar fluids (BALFs) and 60 throat washing (TW) samples with confirmed PCR results, as well as eight throat washes invalid by PCR, were tested with the BIOCREDIT test (RapiGEN), the Panbio^TM^ assay (Abbott), and the SARS-CoV-2 rapid antigen test (Roche). Conclusion: The analyzed antigen test showed an interassay correlation of 27.4%, with overall specificities ranging from 19.4% to 87.1%, while sensitivities of the respective tests ranged between 33.3% and 88.1%. Because these assays did not entirely meet all high expectations, their benefit has to be carefully evaluated for the respective test strategy and setting.

## 1. Introduction

In December 2019, the public became aware of the new betacoronavirus SARS-CoV-2, due to an outbreak in Wuhan, China [1]. Very quickly, it turned out that spreading of the virus could not be prevented and COVID-19 was declared to be a pandemic in March 2020 (https://www.euro.who.int/en/health-topics/health-emergencies/coronavirus-covid-19/news/news/2020/3/who-announces-covid-19-outbreak-a-pandemic). In order to minimize the risk of infection, different undirected, as well as targeted tracking strategies, were developed, and their success was dependent on extensive testing of the highest possible number of people [2]. For SARS-CoV-2 detection, different PCRs are used for routine diagnostics. Although it has been shown that these PCRs actually represent the diagnostic gold standard [3], valuable time passes until the result is available (https://www.nytimes.com/2020/08/04/us/virus-testing-delays.html). Since it has been shown that rapid antibody screenings are not suitable to evaluate chains of infection or their interruption, other kinds of rapid on-site tests are needed to perform the requested mass testing.

Among these are the BIOCREDIT COVID-19 Ag test by RapiGEN (Gyeonggi-do, Korea), the Panbio^TM^ COVID-19 Ag by Abbott (Cologne, Germany), and the SARS-CoV-2 rapid antigen test by Roche (also known as SD Biosensor, Inc. STANDARD Q, Mannheim, Germany). These lateral flow tests (LFTs) are based on immunochromatography and show the presence of SARS-CoV-2 antigen by a colored test line. Sampling should be performed in the nasopharynx with a supplied swab, but universal and viral transport media (UTM and VTM) are also appropriate, except for the Panbio^TM^ assay. Moreover, it has been shown that specimens other than nasopharyngeal swabs are superior regarding diagnostic sensitivity [4,5]. For this reason, we tested a pilot sample panel of 60 throat washes and 13 bronchoalveolar fluids (BALFs) regarding their test performance.

## 2. Results

All samples used in this study had been characterized previously with the Altona two target PCR assay as a gold standard, according to national and international recommendations. This gold standard PCR was successfully validated and certified by analyzing external controls supplied within a national round robin trial organized by INSTAND e.V. and the positive control RNA provided by the German National Reference Centre for coronaviruses. PCR results, in all cases, were used as true positive or true negative for calculations of sensitivity, specificity, and predictive values of lateral flow antigen assays. A total set of 73 samples with valid PCR results were included, plus an additional eight samples with invalid PCR results due to inhibition of the internal control. In total, 11 of 13 BALFs, and 31 of 60 throat washing samples (TWs) were tested as PCR positive.

**Figure 1:**
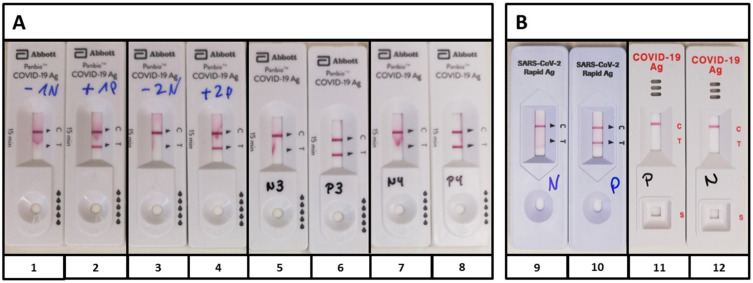
Controls of the Panbio^TM^ COVID-19 Ag test (Abott). (**A**) Supplied positive controls (PCs) (2, 4, 6, 8) and negative controls (NCs) (1, 3, 5, 7) of four kits were performed, according to the manufacturer’s protocol; (**B**) Controls of approach (A) were pooled and applied to the antigen tests by Roche (9, 10) and RapiGEN (11, 12). While the PC by Abbott also became positive with Roche (10), the BIOCREDIT test did not recognize the positive control (11). PC, positive control; NC, negative control.

First, proper performance of the Panbio^TM^ was confirmed with the supplied controls (Figure 1A). After pooling, the Panbio^TM^ controls were applied to the devices by Roche, which also detected the positive control, and by RapiGEN, which was negative for the Abbott Panbio^TM^ controls (Figure 1B). There were no controls delivered with the test devices by Roche and RapiGEN.

**Figure 2:**
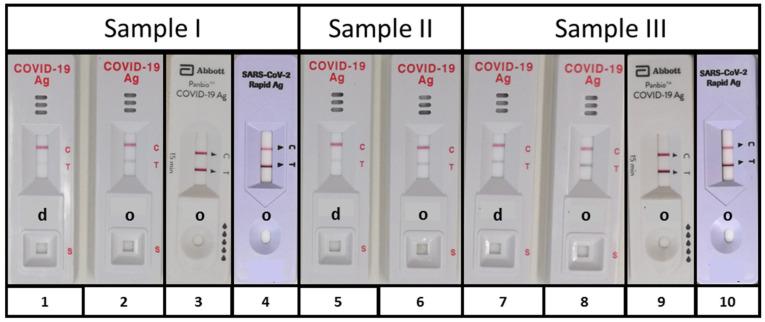
SARS-CoV-2 antigen detection. To evaluate the suitability of specimen others than nasopharyngeal swabs, two PCR positive throat washes, i.e., (Sample I) Ct_E-Gen_ = 15.6 and Ct_S-Gen_ = 14.8 and (Sample II) CT_E-Gen_ = 14.7 and Ct_S-Gen_ = 14.9, and one BALF, i.e., (Sample III) Ct_E-gen_ = 13.1 and Ct_S-Gen_ = 12.6 were used for initial evaluation of lateral flow test performance. SARS-CoV-2 antigen was detected with the BIOCREDIT test in all samples, but dilution in assay buffer (d 1, 5, and 7) decreased sensitivity as compared with original fluid (o 2, 6, and 8). Undiluted samples I and III were also used for an initial analysis with the Panbio^TM^ COVID-19 Ag test (Abott) (3 and 9) and the SARS-CoV-2 rapid antigen test (Roche) (4 and 10) also delivering positive results in both tests. As shown by the control line, the specimens allowed a proper test performance and did not contain any inhibitory substances. d, 1:2 diluted with supplied buffer and o, original.

In order to determine if SARS-CoV-2 antigen could be detected in specimens other than nasopharyngeal swabs, we applied either a diluted or an original PCR positive specimen on the device and checked for the presence of the control line (C) and the test line (T). To increase the probability of antigen detection, samples with Ct values <16 for E- and S-gene were used. Although there was not sufficient material of sample II, this pilot approach showed that the specimens used allowed a proper test performance and that the SARS-CoV-2 antigen could principally be detected in throat washes (Samples I and II) and BALF (Sample III) (Figure 2).

The analysis of the complete test cohort, based on PCR as the gold standard, revealed overall sensitivities of 33.3% (RapiGEN) (95% CI 21% to 48%), 50% (Abbott) (95% CI 35% to 64%), and 88.1% (Roche) (95% CI 75% to 95%) with opposite overall specificities of 87.1% (RapiGEN) (95% CI 71% to 95%), 77.4% (Abbott) (95% CI 60% to 89%), and 19.4% (Roche) (95% CI: 9% to 36%) (Table 1). This means positive predictive values (PPVs) ranging from 59.7% (Roche) (95% CI 89% to 100%) to 77.8% (RapiGEN) (95% CI 94% to 100%) and negative predictive values (NPVs) of 53.3% (Abbott) (95% CI 45% to 92%), 54.6% (Roche) (95% CI 20% to 100%), and 49.1% (RapiGEN) (95% CI 59% to 99%).

For 50 of the 60 TW samples, information on symptomatic state or asymptomatic state was available. The analysis of the 50 samples that were confirmed symptomatic (46%) or asymptomatic (54%) with regard to the respective PCR result, led to sensitivity/specificity values in asymptomatic individuals of 30.8/92.9% (RapiGEN), 38.5/71.4% (Abbott), and 84.6/14.3% (Roche) and to sensitivity/specificity values in symptomatic individuals of 30.0/76.9% (RapiGEN), 40.0/84.6% (Abbott), and 100/7.7% (Roche). The low specificity of the Roche assay is based on the number of positive detections of PCR negative samples in both groups. While 14 samples of asymptomatic and 13 samples of symptomatic individuals were PCR negative, Roche identified 12 of these as positive, respectively. Regarding the BALF specimens, the assays by Abbott and Roche showed performance data of 72.7% or 81.8% sensitivity and 100% specificity, whereas RapiGEN also showed 100% specificity but 54.6% sensitivity (Table 1). This relatively high concordance among the Roche and the Abbott assays and the PCR, respectively, is probably due to the absence of inhibitory agents putatively present in the throat washes or swabs but not present in the BALF.

When comparing the antigen tests with regard to their correlation, it turned out that the RapiGEN-Roche coincidence was 35.6% (26 samples), the RapiGEN-Abbott coincidence was 64.4% (47 samples), and the Roche-Abbott coincidence was 50.7% (37 samples). In total, the LFTs provided the same results in 20 out of 73 samples (27.4%). These results damped the expectation that PCR invalid samples could reliably be analyzed with any of these rapid antigen tests, especially as two of these samples (n = 8) still remained invalid in one LFT, respectively, and only three samples coincided as negative in all LFTs.

**Figure 3:**
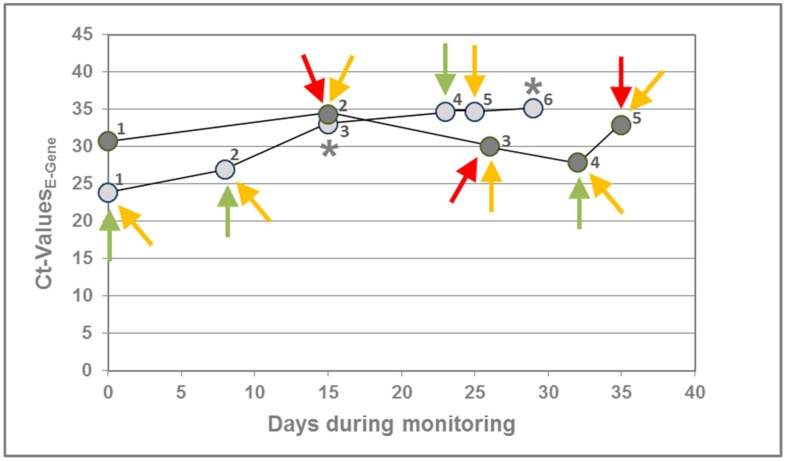
Correlation of SARS-CoV-2 antigen detection with RNA load. This figure shows longitudinal SARS-CoV-2 detection by PCR in two asymptomatic individuals (patient A, dark grey and patient B, light grey). In both cases, SARS-CoV-2 RNA could be detected for about five weeks before recovering in week six. The RapiGEN assay (red) detected SARS-CoV-2 antigen only for patient A in three samples with Ct > 30. Although the assays by Abbott (green) and Roche (yellow) identified 4 and 7 samples as positive, respectively, one sample remained antigen negative (Sample 1 of patient A). Except for the negative sample, there was no coincidence between the LFTs at all. * Only tested negative by RapiGEN, due to insufficient amount of specimen left for the assay.

When checking any correlation of viral RNA load and the presence of SARS-CoV-2 antigen, we were able to monitor two asymptomatic individuals, who were PCR positive for more than five weeks before recovering in week six. The Ct values ranged from 27.9 to 34.3 in patient A (data series dark grey) and from 23.8 to 35.2 in patient B (data series light grey). The RapiGEN assay detected SARS-CoV-2 antigen only in three samples of patient A with Ct values >30 (Figure 3). Sample 4 of patient B was only detected by the Abbott Panbio^TM^ assay and Sample 5 only by the SARS-CoV-2 rapid antigen test by Roche. Sample 1 of patient A remained antigen negative in all tests, despite a positive PCR result, whereas none of the samples was identified as positive in all three assays. However, it appears that based on the data obtained for these two patients, the RapiGEN assays are less efficient than the other assays, although a higher case follow-up series would be required to confirm this assumption.

## 3. Discussion

Due to the pandemic spread of SARS-CoV-2, it would be desirable to possess reliable rapid tests for infection control with early notification of cases to enable effective outbreak management. Therefore, research and development efforts have been focused on rapid diagnostic tests including SARS-CoV-2 antigen tests.

Here, we tested SARS-CoV-2 antigen assays by RapiGEN, Abbott, and Roche. According to the manufacturers’ instructions, sampling should be performed with the supplied swabs, however, this test procedure limits test comparability as every swab represents an individual sample even if taken from the same patient. For this reason, and because UTM/VTM is also appropriate, except for the Abbott Panbio^TM^ assay, we evaluated assay performance with BALF and throat washes as these were performed with a physiological solution of NaCl (0.9% w/v), and thus did not contain any additional interfering chemicals as compared with the allowed transport media. Although the Abbott Panbio^TM^ assay excludes specimens other than swabs, it is unlikely that the Abbott Panbio^TM^ buffer components ProClin300 and sodium acid (preservatives) or tricin (buffer substance capturing divalent metal ions) have direct influence on SARS-CoV-2 antigen binding capacity. Solely missing Tween 20 might have influenced antigen binding in the Abbott PanBio^TM^ assay, and although test control lines occurred properly in all PCR pretested samples, the overall correlation between all LFTs was only 27.4%.

The fact that the positive control supplied by Abbott was detected by Roche, but not by RapiGEN, suggests that the assays may detect different antigens, which additionally complicates the estimation of test comparability and usability in clinical routine settings. Especially, it still remains unclear if further common phenomena such as defective interfering particles, antigen drift, or antigen shift, occurring during the current pandemic, influence assay performance of any SARS-CoV-2 antigen test.

Because SARS-CoV-2 antigen is detected in samples of individuals ranging from asymptomatic + PCR negative to symptomatic + PCR negative, to asymptomatic + PCR positive, and to symptomatic + PCR positive, antigen tests are not suitable for routine diagnostics as long the complex relationships among viral RNA load, SARS-CoV-2 antigen detection, and clinical symptoms remain unsolved. This conclusion is supported by the manufacturers’ recommendations, who explicitly claim that their assays are not approved as a stand-alone diagnostic and the limitations that the assay should be performed “in patients with clinical symptoms” (RapiGEN) or that the test “is not intended to detect from defective (non-infectious) virus during the later stages of viral shedding that might be detected by PCR molecular tests” (Abbott). This means that these assays are not appropriate for screening of asymptomatic individuals. In addition, to date, they cannot be recommended for broad use in any setting in which reliable diagnostics are crucial to avoid spreading of the virus, such as hospitals and long-term care facilities for the elderly or other risk groups, especially, as limited information on host and viral factors influencing shedding of SARS-CoV-2 antigens and their correlation to infectious viruses impede any prognosis on infectivity.

During the review process of this manuscript, further studies were published that also investigated the utility of lateral flow antigen assays for the detection of SARS-CoV-2 and reported similar shortcomings in the overall performance of those assays. Lindner et al. reported sensitivities of 74.4 to 79.5% in agreement with PCR assays, which meant that up to 25% positive cases were not detected by lateral flow assays [6]. Additionally, Weitzel and coworkers evaluated the usage of universal transport medium and its performance on lateral flow antigen devices [7], and revealed that sensitivities ranging from 16.7 to 85% depended on the subgroup of specimens tested. The overall test concordance ranging from 50 to 67% led to the conclusion that the analyzed assays had significant heterogeneity and were solely reliable if specimens with high viral loads were tested [7]. This message was supported by Yamayoshi et al., who reported that many assays did not detect lower virus amounts and that even in cases in which the virus was successfully isolated by cell culture the assays were negative [8]. Moreover, Krüttgen et al. found that the sensitivity of the Roche rapid antigen assay had a serious drop in sensitivity, i.e., as low as 44.8% and lower if the PCR Ct-values were >30, thus, leading to the conclusion that the lateral flow devices were only useful for patients with high viral loads [9]. A low-test performance was observed with the Abbott assay when saliva and nasal samples of asymptomatic patients were applied, which confirmed uncertainties regarding the rapid antigen assay’s suitability for screening purposes [10].

Consequently, although it actually seems that exclusive antigen tests have research character rather than being true in vitro diagnostics (IVDs) and unfortunately cannot replace PCR assays, they should additionally be used to gain deeper insights into infectivity and the course of infection to develop more advanced testing strategies. Moreover, it is likely that the supposed safety reached by performing these rapid antigen assays contributes to the (re-)increasing number of newly detected cases.

## 4. Materials and Methods 

A pilot sample panel of 13 BALFs and 60 throat washing (TW) samples with confirmed PCR results (RealStar^®^ SARS-CoV-2 RT-PCR Kit, Altona, Germany), as well as 8 throat washes invalid by PCR were tested with three SARS-CoV-2 antigen lateral flow tests (LFTs). We compared the BIOCREDIT assay of RapiGen, the Panbio^TM^ assay by Abbott, and the SARS-CoV-2 rapid antigen test by Roche. If supplied, controls were performed according to the manufacturer. For the test procedure, sample volumes of 55 µl (Roche), 110 µl (Abbott), and 150 µl (RapiGen) BALF/TW, instead of extracted swab material, were applied to the respective assays, otherwise tests were performed according to the manufacturers’ recommendations. Additional analysis with the above listed tests were performed with PCR invalid samples (n = 8), thereby invalid means inhibited, i.e., the internal extraction and amplification control of the PCR assay was not detected. All methods and procedures were in accordance with all relevant guidelines and approval from the ethical committee of the Private University of Witten/Herdecke (approval 122/2016). The approval explicitly allowed waiving written informed consent, if full anonymization was warranted and there was an emergence of a pathogen’s outbreak such as observed in the case of COVID-19. Only the corresponding author had access to identifying patient information.

## Figures and Tables

**Table 1:** Sensitivity * and specificity * of SARS-CoV-2 antigen detection in PCR tested specimen.

	RapiGEN		Abbott		Roche	
	Sensitivity	Specificity	Sensitivity	Specificity	Sensitivity	Specificity
BAL	54.6	100	72.7	100	72.7	100
N = 13	(28–78%)	(34–100%)	(43–90%)	(34–100%)	(52–95%)	(34–100%)
TW_symptomatic_	30.0	76.9	40.0	84.6	100	7.7
N = 23	(11–60%)	(50–92%)	(17–69%)	(58-96%)	(72–100%)	(1–33%)
TW_asymptomatic_	30.8	92.9	38.5	71.4	84.6	14.3
N = 27	(13–58%)	(69–99%)	(18–64%)	(45–88%)	(57–96%)	(4–100%)
Total	33.3	87.1	50.0	77.4	88.1	19.4
N = 73	(21–48%)	(71–95%)	(35–64%)	(60–89%)	(75–95%)	(9–36%)

Three lateral flow tests (LFTs) were used for evaluation. Analysis of the complete test cohort (n = 73) reveals overall sensitivities of 33.3% (RapiGEN), 50.0% (Abbott), and 88.1% (Roche). Overall specificities range from 19.4% (Roche) to 77.42% (Abbott), up to 87.1% (RapiGEN). The 50 samples confirmed symptomatic or asymptomatic reveal the highest sensitivities (84.6% and 100%) but poor specificities with Roche, whereas the highest specificities (76.9% and 92.9%) are reached by RapiGEN with sensitivities of 30%, respectively. In total, the three LFTs match in 20 of 73 samples (27.4%). * In percent, BAL, bronchoalveolar lavage and TW, throat washing. In brackets, 95% confidence interval.

## Data Availability

All data are included in the manuscript.
